# A Convolutional-Transformer Residual Network for Channel Estimation in Intelligent Reflective Surface Aided MIMO Systems

**DOI:** 10.3390/s25195959

**Published:** 2025-09-25

**Authors:** Qingying Wu, Junqi Bao, Hui Xu, Benjamin K. Ng, Chan-Tong Lam, Sio-Kei Im

**Affiliations:** Faculty of Applied Sciences, Macao Polytechnic University, Macao SAR, China; qingying.wu@mpu.edu.mo (Q.W.); junqi.bao@mpu.edu.mo (J.B.); hui.xu@mpu.edu.mo (H.X.); ctlam@mpu.edu.mo (C.-T.L.); marcusim@mpu.edu.mo (S.-K.I.)

**Keywords:** reconfigurable intelligent surface, multiple-input multiple-output, channel estimation

## Abstract

Intelligent Reflective Surface (IRS)-aided Multiple-Input Multiple-Output (MIMO) systems have emerged as a promising solution to enhance spectral and energy efficiency in future wireless communications. However, accurate channel estimation remains a key challenge due to the passive nature and high dimensionality of IRS channels. This paper proposes a lightweight hybrid framework for cascaded channel estimation by combining a physics-based Bilinear Alternating Least Squares (BALS) algorithm with a deep neural network named ConvTrans-ResNet. The network integrates convolutional embeddings and Transformer modules within a residual learning architecture to exploit both local and global spatial features effectively while ensuring training stability. A series of ablation studies is conducted to optimize architectural components, resulting in a compact configuration with low parameter count and computational complexity. Extensive simulations demonstrate that the proposed method significantly outperforms state-of-the-art neural models such as HA02, ReEsNet, and InterpResNet across a wide range of SNR levels and IRS element sizes in terms of the Normalized Mean Squared Error (NMSE). Compared to existing solutions, our method achieves better estimation accuracy with improved efficiency, making it suitable for practical deployment in IRS-aided systems.

## 1. Introduction

The Intelligent Reflective Surface (IRS) has emerged as a promising technology for future wireless networks due to its ability to reconfigure the wireless propagation environment in a programmable manner. An IRS consists of a large number of low-cost passive reflective elements, each of which can independently adjust the phase of incident electromagnetic waves [1]. This unique capability enables the IRS to enhance signal coverage, suppress interference, and improve both spectral and energy efficiency without requiring additional active transmitters or power sources [2,3,4]. When deployed in Multiple-Input Multiple-Output (MIMO) systems, an IRS can relieve Non-Line-of-Sight (NLoS) communication losses by establishing virtual links between the User Terminal (UT) and Base Station (BS) even in the presence of tall structures or other obstacles [5,6].

Despite its promising potential in wireless communication systems, the IRS presents several technical challenges. A major challenge is that the performance of IRS-aided systems critically depends on the availability of accurate Channel State Information (CSI) [7], which is essential for subsequent tasks such as beamforming, user scheduling, and rate adaptation. Unlike conventional MIMO systems that rely on active radio-frequency chains for signal transmission and reception, IRS elements are inherently passive and incapable of performing independent communication [8]. This leads to the high dimensionality of the cascaded BS-IRS-UT channel, along with the fact that the pilot overhead scales with the number of IRS elements, and further causes a trade-off between the accuracy of the estimate and the cost of signaling [9,10,11].

Beyond RIS-specific designs, the broader wireless communication community has explored various advanced signal processing and resource optimization methods to improve channel utilization, spectral efficiency, and energy harvesting capabilities. For example, faster-than-Nyquist-assisted simultaneous wireless information and power transfer-nonorthogonal multiple access schemes have been proposed to simultaneously enhance wireless efficiency and energy harvesting performance in IoT networks [12], while adaptive damping message-passing algorithms have been introduced for faster-than-Nyquist orthogonal time frequency space systems to improve robustness in high-mobility vehicle-to-everything communications [13]. Although these approaches are not directly applied to RIS scenarios, they represent important progress in non-deep learning-based channel processing and provide valuable insights for designing efficient algorithms in emerging wireless architectures. In the context of IRS-aided systems, the Bilinear Alternating Least Squares (BALS) method models the received signal via a parallel factor (PARAFAC) tensor decomposition to estimate the cascaded channel efficiently [14].

More recently, deep learning (DL)-based approaches have been applied to leverage channel reciprocity. By using uplink CSI to estimate downlink CSI, these approaches achieve notable gains in estimation accuracy [15,16,17,18]. Despite the progress made by prior works, existing methods still face several limitations. Many of these methods rely on deep structures with large parameter counts, resulting in increased computational burden and reduced suitability for real-time applications. Additionally, most existing designs emphasize either convolutional or attention-based mechanisms, without fully leveraging the complementary strengths of both. DL models also remain vulnerable to training instabilities such as gradient vanishing and model degradation, particularly when stacking multiple layers. These challenges motivate the development of a lightweight yet expressive channel estimation framework.

In this paper, we propose a Convolutional-Transformer Residual Network (ConvTrans-ResNet) for efficient channel estimation in RIS-aided MIMO systems. The model operates in two stages. First, a coarse but structured estimate of the cascaded channel is obtained using the BALS algorithm. This estimate is then denoised using the proposed method to capture both local and global spatial dependencies. Compared with existing methods, the proposed design ensures low complexity, stable training, and strong generalization across a wide range of scenarios. The main contributions of this work can be summarized as follows.

- We propose a lightweight neural network for cascaded channel estimation in RIS-aided MIMO systems. The model denoises a coarse channel estimate obtained with the BALS algorithm so that both model-driven interpretability and data-driven representation are combined to enhance estimation accuracy.
- To fully exploit the structured nature of RIS channels, the proposed network incorporates convolutional embedding blocks for extracting fine-grained local spatial features and Transformer modules for capturing long-range dependencies. This complementary design addresses the limitations of existing methods that rely solely on either convolutional or attention-based architectures.
- To ensure training stability and relieve gradient degradation, a residual learning framework is adopted. This design facilitates gradient flow, accelerates convergence, and improves robustness against noise, thereby guaranteeing effective deep stacking without sacrificing accuracy.
- To systematically investigate the impact of key architectural parameters and further identify an optimal configuration, a detailed ablation study is conducted. Simulation results also demonstrate that the proposed ConvTrans-ResNet consistently outperforms state-of-the-art approaches such as ReEsNet, InterpResNet, HA02, and BALS across a wide range of SNRs and IRS sizes, while significantly reducing parameter count and FLOPs, making it highly suitable for real-time and resource-constrained deployments.

The remainder of this paper is structured as follows. Recent works on channel estimations in RIS-aided systems are summarized in Section 2. In Section 3, we introduce the considered IRS-aided MIMO system. The existing BALS method is explained in Section 4. In Section 5, the proposed ConvTrans-ResNet is detailed. To evaluate the effectiveness of our proposed method, the simulation results and analysis are presented in Section 6. Finally, the paper is concluded in Section 7.

*Notation:* In this paper, the transpose and pseudo-inverse of a matrix A are denoted as $A^{T}$ and $A^{†}$, respectively. In addition, ⋄ denotes the Khatri Rao and $I_{N}$ denotes the N×N identity matrix.

## 2. Related Work

Numerous methods have been proposed in recent years to address the channel estimation challenge. To reduce pilot overhead, [19] has leveraged the observation that each user shares a uniform IRS-to-BS link, thereby significantly reducing the required pilot length. In [14], a tensor decomposition framework was introduced, linking IRS-aided MIMO communications with the PARAFAC model. By designing structured time-domain pilot and phase shift patterns, the received signal conforms to a PARAFAC tensor model. This formulation enables the use of the BALS algorithm for efficient cascaded channel estimation. As a physics-informed iterative method, BALS exploits the bilinear channel structure and is particularly well-suited to block-fading scenarios, where the channel remains constant over multiple training intervals.

Advances in DL have also contributed to channel estimation research. Tabassum et al. [20] designed recurrent neural network-based models that decode transmitted symbols directly from received OFDM signals, bypassing explicit CSI estimation in favor of end-to-end learning. Ye et al. [21] introduced a conditional generative adversarial network to directly estimate the channel from pilot sequences and observation signals in IRS-aided systems. In [18], to denoise channel estimation, a convolutional deep residual network was used in IRS multi-user communication systems. Similarly, a twin Convolutional Neural Network (CNN) architecture was proposed to estimate both direct (BS-UE) and cascaded (BS-IRS-UE) channels in millimeter-wave massive MIMO systems, as discussed in [22]. For the frequency division duplex-based Massive MIMO systems, Abdelmaksoud et al. [23] proposed a denoising gated recurrent unit with dropout-based CSI extraction, but this method does not leverage the inherent low-rank structure of IRS channels, with generalization capabilities limited. In contrast, Janawade et al. [24] employed reinforcement learning to directly optimize IRS phase configurations without estimating the channel. While efficient, this approach lacks flexibility for tasks requiring full CSI.

Despite their advantages, neural network-based solutions often face issues such as gradient vanishing and model degeneration. To address it, Li et al. [25] presented the Residual Channel Estimation Network (ReEsNet), a residual CNN that offers improved efficiency and reduced complexity. An improved deep residual shrinkage network was also introduced in [15] to improve the pilot design by effectively reducing noise, making it advantageous in stable channel conditions. The authors in [16] used a residual U-shaped network and a deep CS-based channel estimation model to identify the cascaded channel matrix with minimal pilot overhead.

Some studies have shown that combining model-based signal processing with deep learning can significantly improve channel estimation accuracy and ensure robustness. For instance, researchers presented a hybrid IRS structure and DL-based CNN for sparse channel amplitude determination in [17]. However, the added complexity of the model may decrease channel estimation accuracy, making it less suitable for real-time applications. Luan and Thompson [26] proposed a hybrid encoder–decoder network (HA02) that uses a transformer encoder to extract salient features from least-square estimates, followed by a residual convolutional decoder for channel denoising in the orthogonal frequency-division multiplexing system. This design leverages the attention mechanism to selectively emphasize important components of the input, thereby enhancing performance and robustness. Similarly, Gu et al. [27] introduced ReEsNet and InterpResNet channel estimation networks built on preliminary BALS estimates of the cascaded channel. Their approach demonstrates that a data-driven network can effectively denoise coarse physics-based estimates and generalize across different numbers of IRS elements. These studies confirm the advantages of hybrid frameworks: The traditional algorithm provides domain-aware initialization, while the deep network performs non-linear denoising and structural interpolation, leading to improved robustness, better generalization, and reduced training complexity.

Several recent works have further advanced IRS-aided channel estimation. Chu et al. proposed an adaptive and robust framework for mmWave systems, where a parallel estimation strategy mitigates error propagation and improves robustness [28]. An automatic neural network construction method was introduced that uses neural architecture search to generate high-performance channel estimators tailored to specific propagation conditions in [29]. In [30], active IRS-aided IoT systems were investigated, where joint power optimization and deep learning techniques improve channel estimation under stringent power budgets. The study on joint location sensing and channel estimation for IRS-aided mmWave ISAC systems demonstrates that structured sparsity and Bayesian inference can support integrated communication and sensing [31]. The power measurement-based estimation scheme was also developed to reduce pilot overhead by leveraging received signal power and lightweight neural networks [32]. These recent contributions highlight the rapid progress in IRS-aided channel estimation, covering robust optimization, neural architecture design, and integration with IoT and ISAC applications.

## 3. System Model

**Figure 1:**
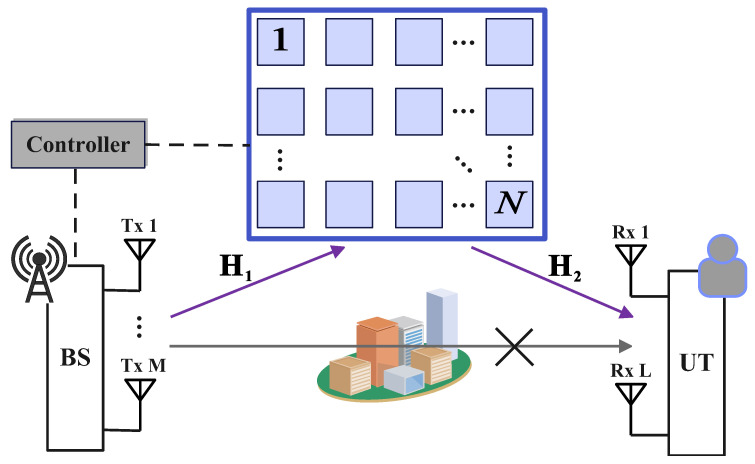
Demonstration of an IRS-aided MIMO system. There are *M* and *L* antennas equipped at the BS and UT, respectively. The IRS comprises *N* passive elements and reflects signals from the BS to the UT under the control of a centralized controller. The direct LoS path is obstructed.

As depicted in Figure 1, we consider a downlink communication scenario in an IRS-aided MIMO system. In this setup, the BS is equipped with *M* transmit antennas and communicates with a UT with *L* receive antennas. The communication link between the BS and the UT is established via an IRS, which consists of *N* passive elements arranged in a Uniform Planar Array (UPA). Each IRS element can independently adjust the phase of the incident signal, thereby reconfiguring the wireless propagation environment. These phase shifts are controlled through a centralized controller. Notably, we assume that the direct Line-of-Sight (LoS) path between the BS and the UT is completely blocked due to obstacles in the environment. Therefore, the reflected link via the IRS becomes the only feasible channel for signal transmission.

Assuming a block-fading environment with coherence time $T_{c}$, the received signal at time instant $t\in \left\{1,\ldots ,T_{c}\right\}$ is modeled as

(1) $$y_{t}=H_{2}\left(s_{t}⊙H_{1}x_{t}\right)+n_{t},$$

where $H_{1}\in C^{N\times M}$ and $H_{2}\in C^{L\times N}$ denote the BS-IRS and the IRS-UT channel matrix, respectively. Both $H_{1}$ and $H_{2}$ are assumed to be independent and identically distributed zero-mean circularly symmetric complex Gaussian random variables. The term $n_{t}\in C^{L\times 1}$ represents Additive White Gaussian Noise (AWGN). $x_{t}\in C^{M\times 1}$ denotes the transmitted pilot signal, $s_{t}={\left[s_{1,t}e^{j\phi_{1}},\ldots ,s_{N,t}e^{j\phi_{N}}\right]}^{T}\in C^{N\times 1}$ is the IRS configuration vector, where $s_{n,t}\in \left\{0,1\right\}$ controls the on/off control status of element *n* at time *t* and $\phi_{n}\in \left(0,2\pi \right]$ denotes the phase shift applied by the *n*-th IRS element. For analytical convenience, with the assumption that both $H_{1}$ and $H_{2}$ are independent and identically distributed zero-mean circularly symmetric complex Gaussian random variables, the received signal can be alternatively expressed as [33]

(2) $$y_{t}=H_{2}\mathrm{diag}\left(s_{t}\right)H_{1}x_{t}+n_{t}.$$

**Figure 2:**
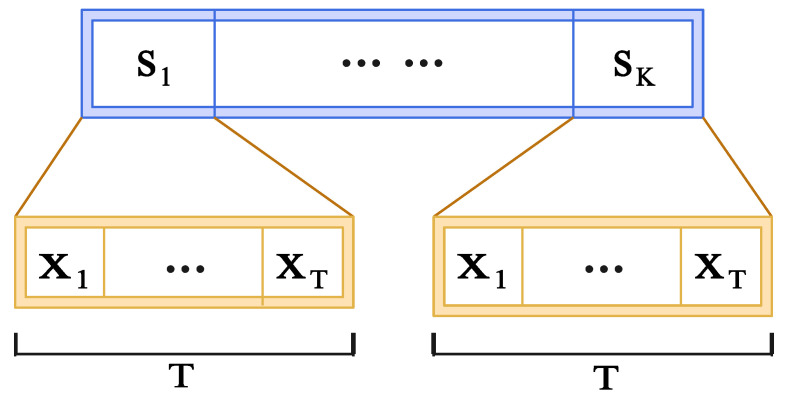
Illustration of the structured pilot transmission strategy in the time domain. Each block $S_{k}$ spans *T* time slots, within which the pilot signals $\left\{x_{1},\ldots ,x_{T}\right\}$ are repeated while the IRS phase shift vector remains fixed.

Let the total coherence interval be $T_{c}=KT$, where *K* denotes the number of blocks, and each block comprises *T* time slots. As illustrated in Figure 2, a structured time-domain protocol is adopted such that the phase shift vector $s_{k}$ remains fixed within each block and varies only across blocks. In contrast, the pilot signal sequence $\left\{x_{1},\ldots ,x_{T}\right\}$ is reused across all *K* blocks. Accordingly, the received signal can be defined as $y_{k,t}≐y\left[(k-1)T+t\right]$, for $t=1,\ldots ,T_{c}$, k=1,…,K. Similarly, the pilot signal and phase shift vectors are defined as

(3) $$\begin{array}{c} x_{k,t}=x_{k},\;\;\mathrm{for}\;t=1,\ldots ,T_{c},\\ s_{k,t}=s_{t},\;\;\mathrm{for}\;k=1,\ldots ,K, \end{array}$$

Thus, the received signal can be simplified to

(4) $$y_{k,t}=H_{2}\mathrm{diag}\left(s_{k}\right)H_{1}x_{t}+n_{k,t}.$$

By aggregating the received signals over *T* time slots into a matrix $Y_{k}=\left[y_{k,1},\ldots ,y_{k,T}\right]$, the block-wise received signal can be written as

(5) $$Y_{k}=H_{2}D_{k}\left(S\right)H_{1}X^{T}+N_{k},$$

where $X≐{\left[x_{1},\ldots ,x_{T}\right]}^{T}\in C^{T\times M}$ is the stacked pilot matrix and $N≐\left[n_{1},\ldots ,n_{T}\right]$ denotes Additive White Gaussian Noise (AWGN). $D_{k}\left(S\right)=\mathrm{diag}\left(s_{k}\right)$ represents a diagonal matrix holding the *k*-th row of the IRS phase shift matrix S on its main diagonal, where $S=\left[s_{1},\ldots ,s_{K}\right]\in C^{K\times N}$ stacks all IRS configurations over blocks.

To further facilitate structured modeling, the noiseless received signal is defined as

(6) $${\bar{Y}}_{k}=H_{2}D_{k}\left(S\right)Z^{T},$$

where $Z=XH_{1}^{T}\in C^{T\times N}$ and ${\bar{Y}}_{k}\in C^{L\times T}$ is the *k*-th frontal matrix slice of a three-way tensor $\bar{Y}\in C^{L\times T\times K}$ that follows a PARAFAC decomposition [34]. At (l,t,k)-th entry, the noiseless received signal tensor is expressed as

(7) $${\left[\bar{Y}\right]}_{l,t,k}=\sum_{n=1}^{N}g_{l,n}z_{t,n}s_{k,n},$$

where $g_{l,n}≐{\left[H_{2}\right]}_{l,n}$, $z_{t,n}≐{\left[Z\right]}_{t,n}$, and $s_{k,n}≐{\left[S\right]}_{k,n}$. This naturally leads to a Canonical Polyadic (CP) decomposition [34,35,36,37,38]: $\bar{Y}=\left[\left[H_{2},Z,S\right]\right]\in C^{L\times T\times K}$. The tensor unfoldings along three modes are given by [34,35]

(8) $${\bar{Y}}_{\left(1\right)}=H_{2}{\left(S⋄Z\right)}^{T}\in C^{L\times TK},$$

(9) $${\bar{Y}}_{\left(2\right)}=Z{\left(S⋄H_{2}\right)}^{T}\in C^{T\times LK},$$

(10) $${\bar{Y}}_{\left(3\right)}=R{\left(Z⋄H_{2}\right)}^{T}\in C^{K\times LT},$$

where ⋄ denotes the Khatri-Rao product and ${\bar{Y}}_{\left(n\right)}$ is the mode-*n* unfolding of tensor $\bar{Y}$. Specifically, ${\bar{Y}}_{\left(1\right)}≐\left[{\bar{Y}}_{1},\ldots ,{\bar{Y}}_{K}\right]$, ${\bar{Y}}_{\left(2\right)}≐\left[{\bar{Y}}_{1}^{T},\ldots ,{\bar{Y}}_{K}^{T}\right]$, and ${\bar{Y}}_{\left(3\right)}≐{\left[\mathrm{vec}\left({\bar{Y}}_{1}\right),\ldots ,\mathrm{vec}\left({\bar{Y}}_{K}\right)\right]}^{T}$.

## 4. Traditional BALS-Based Channel Estimation

To obtain an initial estimate of the cascaded channel, the BALS method has been proposed by exploiting the PARAFAC structure inherent in the received signal tensor. Let $Y≐\bar{Y}+N$ denote the observed signal tensor, where $\bar{Y}$ is the noiseless signal component and $N\in C^{L\times T\times K}$ is the additive noise tensor with independent and identically distributed complex Gaussian entries. Likewise, we can define $Y_{\left(n\right)}≐{\bar{Y}}_{\left(n\right)}+N_{\left(n\right)},n=1,2,3$ as noisy versions of the one-mode, two-mode and three-mode matrix unfoldings in Equations (8)–(10), and $N_{\left(n\right)}$ are the corresponding noise matrix unfoldings.

The tensor decomposition begins with the three mode-*n* unfoldings $Y_{\left(n\right)}$ of Y, which correspond to different dimensions of the tensor. Specifically, we have $Y_{\left(1\right)}\in C^{L\times TK}$, $Y_{\left(2\right)}\in C^{T\times LK}$, $Y_{\left(3\right)}\in C^{K\times LT}$. Among these, the first two unfoldings are primarily used to recover the unknown BS–IRS channel matrix $H_{1}$ and IRS–UT channel matrix $H_{2}$. The pilot matrix X and IRS phase configuration matrix S are assumed to be truncated discrete Fourier transform (DFT) matrices, satisfying the orthonormality conditions $X^{H}X=I_{M}$ and $S^{H}S=I_{N}$.

The BALS algorithm alternates between updating $H_{2}$ and $H_{1}$ by minimizing the following Frobenius-norm-based cost functions:

(11) $$\left\{\begin{array}{c} {\hat{H}}_{1}=\underset{H_{1}}{\arg\min}{∥Y_{\left(2\right)}-XH_{1}^{T}{\left(S⋄H_{2}\right)}^{T}∥}_{F}^{2}\\ {\hat{H}}_{2}=\underset{H_{2}}{\arg\min}{∥Y_{\left(1\right)}-H_{2}{\left(S⋄Z\right)}^{T}∥}_{F}^{2} \end{array}\right.$$

where $Z=XH_{1}^{T}$. These subproblems admit closed-form solutions based on Moore–Penrose pseudoinverses:

(12) $$\left\{\begin{array}{c} {\hat{H}}_{1}^{T}=X^{†}Y_{\left(2\right)}{\left[{\left(S⋄H_{2}\right)}^{T}\right]}^{†}\\ {\hat{H}}_{2}=Y_{\left(1\right)}{\left[{\left(S⋄Z\right)}^{T}\right]}^{†} \end{array}\right.$$

Since both X and S are orthonormal by design, their pseudoinverses can be efficiently computed and, in some cases, replaced by their Hermitian transposes. This significantly reduces the computational complexity of the BALS updates. The detailed procedure of the BALS algorithm is outlined in Algorithm 1.
**Algorithm 1** Bilinear Alternating Least Squares (BALS)1:Initialization: Set iteration index i=0, and random ${\hat{H}}_{1}^{\left(0\right)}$.2:**repeat**3:   i←i+1;4:   Update to find the least square estimate of $H_{2}$ as(13)$${\hat{H}}_{2}^{\left(i\right)}=Y_{\left(1\right)}{\left[{\left(S⋄X{\hat{H}}_{1}^{T(i-1)}\right)}^{T}\right]}^{†}$$5:   Update to find the least square estimate of $H_{1}$ as(14)$${\hat{H}}_{1}^{T(i)}=X^{†}Y_{\left(2\right)}{\left[{\left(S⋄H_{2}^{\left(i\right)}\right)}^{T}\right]}^{†}$$6:**until** convergence.

At each iteration *i*, the reconstruction error is evaluated as $e\left(i\right)={∥Y-{\hat{Y}}_{\left(i\right)}∥}_{F}^{2}$ until it terminates when the error falls below a predefined threshold $10^{-6}$. Once the iterative process converges, the preliminary estimate of the cascaded channel ${\hat{H}}_{c}={\hat{H}}_{2}{\hat{H}}_{1}^{T}$ can be obtained. In this study, the estimate serves as the initial channel representation for further denoising in the IRS-aided MIMO system.

It is important to note that although BALS alternately updates $H_{1}$ and $H_{2}$, both sub-channels are refined within the same iterative optimization loop. Therefore, BALS does not incur irreversible stage-to-stage error propagation. Any bias introduced in one update can be corrected in subsequent iterations, and the final cascaded channel estimate ${\hat{H}}_{c}$ can serve as a stable initialization for the proposed denoising network.

## 5. Proposed Method

### 5.1. Overview

Unlike conventional methods that primarily utilize stacked convolutional blocks to model local features, our approach enhances estimation accuracy by incorporating global spatial correlations through attention mechanisms. Specifically, we propose a lightweight Transformer-based network for IRS-aided MIMO systems, which is capable of preserving structural characteristics in complex propagation scenarios. The proposed method directly targets the cascaded channel estimate ${\hat{H}}_{c}$, which is inherently free from error propagation between sub-channel estimates.

**Figure 3:**
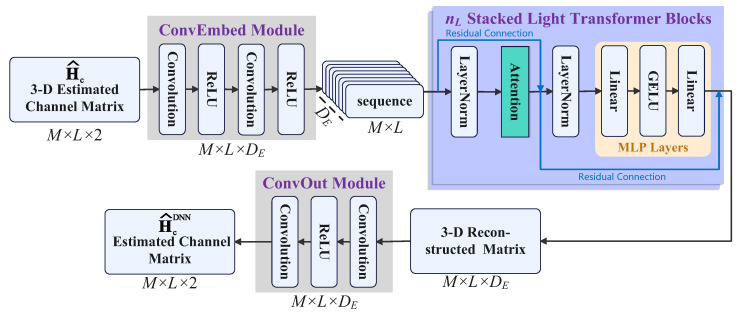
Illustration of the proposed ConvTrans-ResNet architecture. The model denoises the coarse channel estimate $\hat{H_{c}}\in R^{M\times L\times 2}$ using 3 main components: ConvEmbed module for local feature extraction and dimensional lifting, stacked Transformer blocks with residual connections for modeling spatial dependencies, and ConvOut module for channel reconstruction.

As illustrated in Figure 3, the proposed ConvTrans-ResNet consists of three main components: a convolutional embedding (ConvEmbed) module, a residual Transformer encoder composed of multiple stacked attention blocks, and a convolutional output (ConvOut) module. The model denoises the coarse channel estimate $\hat{H}$ by transforming it through convolutional encoding, self-attention-based denoising, and reconstruction. This architecture is designed to capture both local and global spatial dependencies while maintaining low complexity and stable training dynamics.

### 5.2. ConvEmbed Module

In our design, the ConvEmbed module serves as the feature extractor to capture local spatial patterns from the input channel tensor $X\in R^{M\times L\times 2}$. It consists of two sequential 3×3 convolutional layers, each followed by ReLU activation. Apart from projecting the input into a higher-dimensional feature space of size $D_{E}$, the module enhances the expressiveness of local features while preserving fine-grained spatial resolution. The output feature map $F\in R^{M\times L\times D_{E}}$ is then reshaped into $D_{E}$ sequences of shape $R^{M\times L}$ so that it is compatible with the Transformer module.

### 5.3. Transformer Module with Residual Connections

To capture both fine-grained and long-range dependencies, the sequence output from the ConvEmbed module is processed by $n_{L}$ stacked Transformer encoder blocks. Each block consists of a Multi-Head Self-Attention (MHSA) mechanism and a feed-forward multilayer perceptron (MLP), both equipped with residual connections.

Given an input sequence $S\in R^{M\times L\times D_{E}}$, the scaled dot-product attention for each head is defined as

(15) $$\mathrm{Attention}\left(Q,K,V\right)=\mathrm{softmax}\left(\frac{QK^{T}}{\sqrt{d_{k}}}\right)V,$$

where the query (*Q*), key(*K*), and value (*V*) matrices are computed via

(16) $$\left\{\begin{array}{c} Q=XW^{Q},\\ K=XW^{K},\\ V=XW^{V}. \end{array}\right.$$

with $W^{Q},W^{K},W^{V}\in R^{D_{E}\times d_{k}}$ denoting learnable projection matrices and $d_{k}=D_{E}/n_{H}$. The outputs from all heads are concatenated and linearly projected as

(17) $$\mathrm{MHSA}\left(S\right)=\mathrm{Concat}\left({\mathrm{head}}_{1},\ldots ,\mathrm{head}n_{H}\right)W^{O},$$

where $W^{O}\in R^{D_{E}\times D_{E}}$ is a trainable projection matrix. With multiple attention heads, complementary perspectives on the channel representation can be provided. Each head learns a different projection of the input features, which enables the model to capture diverse types of spatial dependencies. For instance, some heads may emphasize broad global correlations across the entire channel matrix, while others focus on localized structures associated with dominant paths or clusters. By jointly aggregating these heterogeneous patterns, the multi-head mechanism enriches the representation capacity of the Transformer module to help denoise coarse channel matrices.

Each Transformer block also includes an MLP sublayer composed of two linear layers and a GELU activation, which can be expressed as

(18) $$\mathrm{MLP}\left(x\right)=W_{2}\left(\mathrm{GELU}\left(W_{1}x\right)\right)+b_{2},$$

where $W_{1}\in R^{D_{E}\times rD_{E}}$ and $W_{2}\in R^{rD_{E}\times D_{E}}$, with *r* denoting the MLP expansion ratio.

Residual connections are applied after both the self-attention and MLP sublayers, followed by layer normalization. These residual paths play a crucial role in maintaining information flow and training stability, particularly in deeper Transformer architectures. Specifically, the residual connection after the self-attention module allows the network to retain the original input features, which is beneficial when the attention weights are sparse or uncertain. This ensures that the model does not lose critical positional or contextual information. The residual link after the MLP block helps mitigate the problem of vanishing gradients by providing a direct path for gradient backpropagation. It also prevents the degradation of learned representations during deep stacking, thereby enabling more effective convergence and better generalization. Together, these mechanisms ensure that the Transformer module can robustly model both fine-grained and global channel structures in RIS-aided MIMO systems.

### 5.4. ConvOut Module

After attention processing, the feature sequence is reshaped back to the spatial format $R^{M\times L\times D_{E}}$. The ConvOut module then denoises this representation through two successive 3×3 convolutions with ReLU activations. More than reducing the feature dimension to match the channel output format, this module also fuses contextual information aggregated by the previous Transformer module. Specifically, the ConvOut module enhances local consistency and ensures that the final output retains high-resolution structural details by applying convolutions over spatial dimensions. The last convolutional layer projects the feature map to a two-channel output, corresponding to the real and imaginary parts of the estimated cascaded channel. This makes the final output compatible with the ground-truth channel format used for supervision during training.

## 6. Experimental Results

To comprehensively assess the performance of the proposed ConvTrans-ResNet model, we conduct comparative experiments against several state-of-the-art channel estimation methods, including ReEsNet [27], InterpResNet [27], HA02 [26], and the baseline BALS algorithm [14]. After detailing the simulation setup, experimental results in terms of estimation accuracy are presented. Furthermore, we analyze the contributions of different architectural components through ablation studies and assess the computational efficiency of the proposed method to ensure its practicality in real-world deployment scenarios.

### 6.1. Implementation Details

Experiments are implemented on 13th Gen Intel(R) Core(TM) i7-13700KF CPU (24 logical CPUs, 32 GB RAM; Intel Corporation, Santa Clara, CA, USA) and an NVIDIA GeForce RTX 3080 GPU with 10 GB VRAM (NVIDIA Corporation, Santa Clara, CA, USA).

#### 6.1.1. Dataset

The dataset is generated based on a block-fading channel model, where the signal-to-noise ratio (SNR) varies from 0 dB to 30 dB in increments of 5 dB. For each SNR level, 5000 independent channel realizations are simulated, i.e., a total of 35,000 samples. Among them, 95% are used for training and the remaining 5% for testing. The simulation parameters are summarized in Table 1. All models are trained and evaluated on the same dataset to ensure a fair and consistent comparison.

#### 6.1.2. Parameters

The mean square error is used as the error function for training ReEsNet, InterpResNet, and the proposed method. HA02 employs the Huber loss, which is defined as

(19) $$L_{\delta }\left(a\right)=\left\{\begin{array}{c} \frac{1}{2}a^{2},\;\;\mathrm{if}\;\left|a\right|\leq \delta ,\\ \delta (\left|a\right|-\frac{1}{2}\delta ),\;\;\mathrm{otherwise}. \end{array}\right.$$

The hyperparameter settings for each method are summarized in Table 2.

#### 6.1.3. Performance Metric

To quantify estimation performance, we adopt the Normalized Mean Squared Error (NMSE) as the performance metric, which is defined as

(20) $$\mathrm{NMSE}=E\left\{\frac{{∥{\hat{H}}_{c}^{\mathrm{DNN}}-H_{c}∥}_{2}^{2}}{{∥H_{c}∥}_{2}^{2}}\right\}$$

where $H_{c}$ and ${\hat{H}}_{c}^{\mathrm{DNN}}$ denote the ground truth and the estimated cascaded channel matrices, respectively. A lower NMSE indicates higher channel estimation accuracy.

### 6.2. Effectiveness Validation

**Figure 4:**
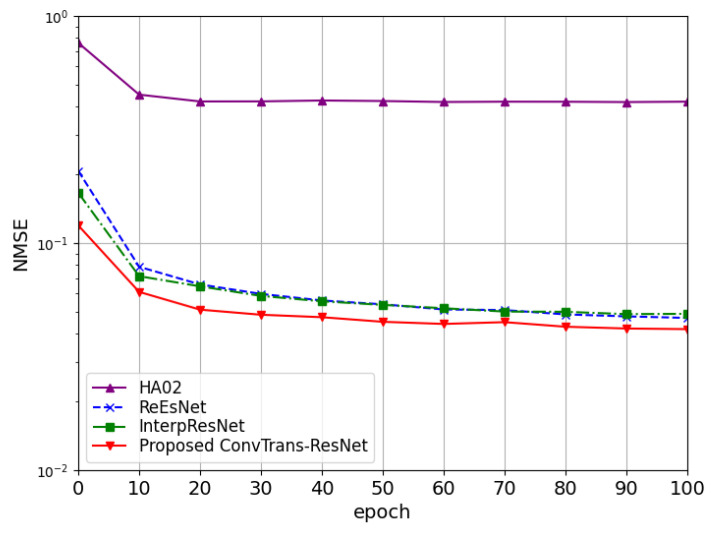
Convergence of various methods in terms of NMSE with N=25 at 0 dB.

To investigate the convergence of different models, Figure 4 presents the NMSE curves during training with 25 IRS elements at 0 dB. The proposed ConvTrans-ResNet converges smoothly and maintains the lowest NMSE across the entire training process. InterpResNet and ReEsNet also converge within 40 epochs, but their final NMSE values remain higher than the proposed method. In contrast, HA02 converges fast in the early stage but performs worst in terms of estimation accuracy.

**Figure 5:**
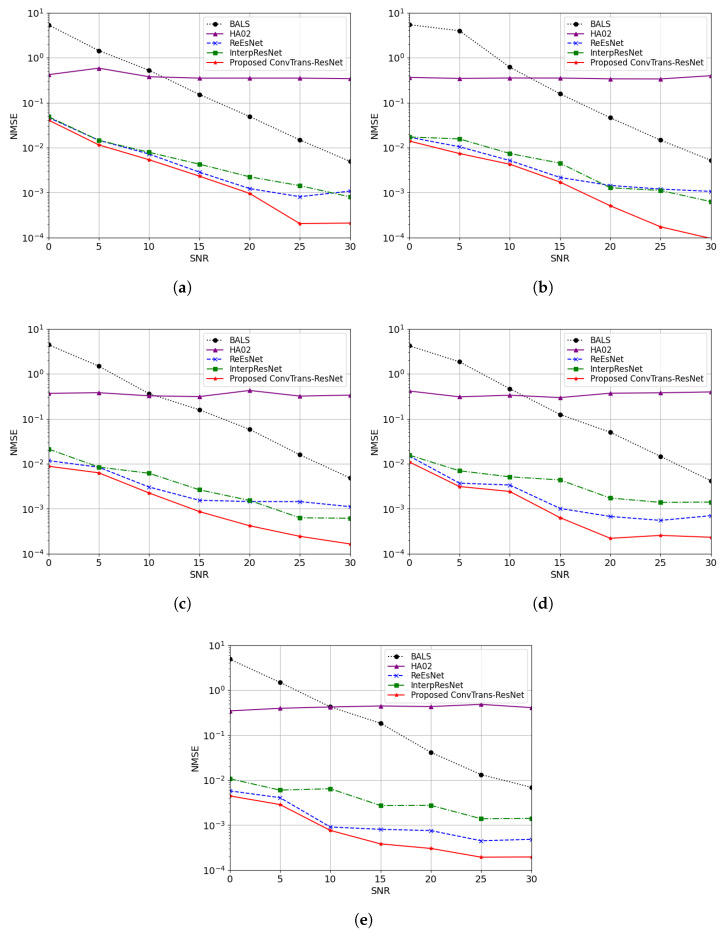
Comparison of different methods in terms of NMSE under SNRs ranging from 0 to 30 dB with various numbers of IRS elements *N*: (**a**) N=25; (**b**) N=49; (**c**) N=81; (**d**) N=100; (**e**) N=144.

Figure 5 illustrates the NMSE performance of different channel estimation methods with various IRS elements under SNRs from 0 dB to 30 dB. It can be observed that all DL-based models significantly outperform the traditional BALS algorithm at lower SNR ranges, which validates the effectiveness of data-driven approaches in modeling complex channel matrices. HA02, which adopts a pure Transformer encoder–decoder architecture without convolutional enhancement, exhibits the highest NMSE among DL-based models. This suggests that the lack of local context modeling limits the performance of attention-based designs. ReEsNet and InterpResNet show competitive performance but suffer from performance degradation at low SNRs. This is likely due to their limited receptive fields, which hinder the modeling of long-range spatial dependencies. Among these methods, the proposed ConvTrans-ResNet consistently achieves the lowest NMSE across all SNR levels, particularly at high-SNR conditions (above 20 dB). The outperformance compared to ReEsNet and InterpResnet further confirms the superior denoising capability and estimation accuracy benefits from convolutional feature extraction and attention-based global modeling.

**Figure 6:**
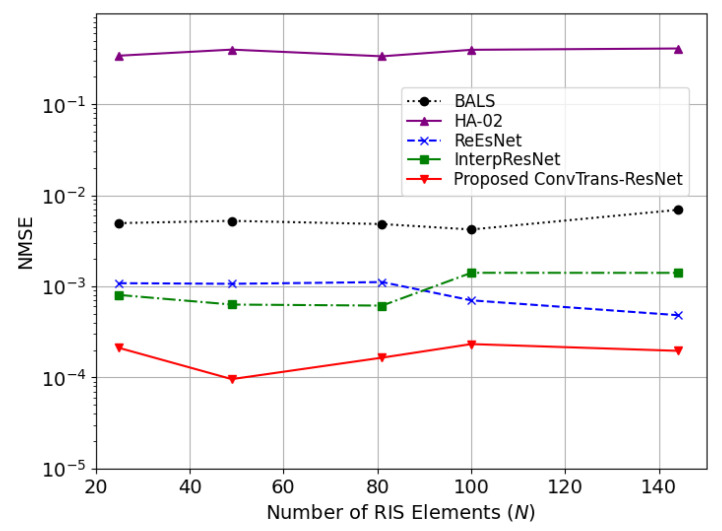
Comparison of various methods in terms of NMSE with various numbers of IRS elements *N* at 30 dB.

Figure 6 presents the NMSE results under different numbers of IRS elements $N\in \left\{25,49,81,100,144\right\}$. The proposed method still performs best across all configurations, indicating its scalability and robustness with an increasing number of IRS elements. As *N* increases, ReEsNet and InterpResNet exhibit slight performance degradation, which reveals the limited generalization when faced with a large-scale IRS deployment. The worst performance of HA02 shows its inadequate adaptability. It is worth noting that while BALS maintains a consistent NMSE trend, it remains significantly less accurate than learning-based methods. As a lightweight and interpretable solution, BALS’s estimation capability is constrained by its reliance on linear algebraic structure, which highlights the necessity of learning-based denoising.

**Figure 7:**
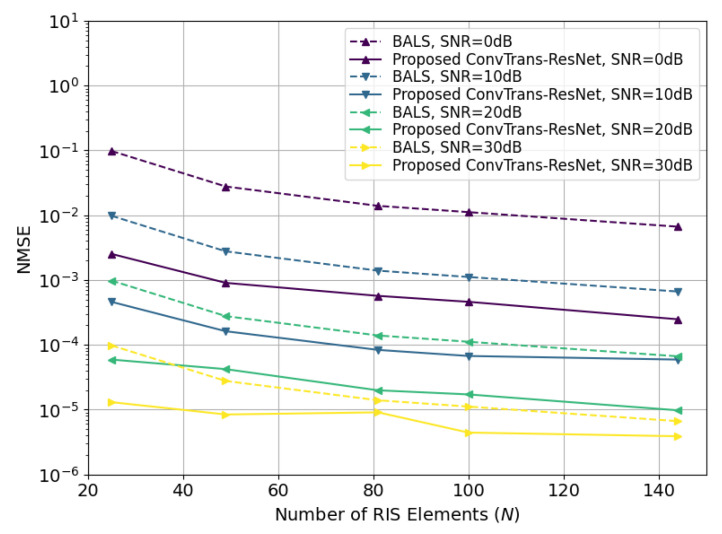
Comparison of BALS and ConvTrans-ResNet in terms of NMSE with varying numbers of IRS elements *N* under the 3GPP channel model.

To further assess generalizability under realistic propagation, we adopt the 3GPP TR 38.901 channel model (urban microcell, NLOS) to generate the BS–IRS and IRS–UE links with pathloss, shadowing, angle spreads, and spatial correlation. As illustrated in Figure 7, the NMSE performance of the proposed ConvTrans-ResNet consistently outperforms BALS across different numbers of RIS elements and various SNR levels under the practical 3GPP channel models. Therefore, the robustness and generalization of the proposed method for real-world IRS-aided MIMO systems can be confirmed.

**Figure 8:**
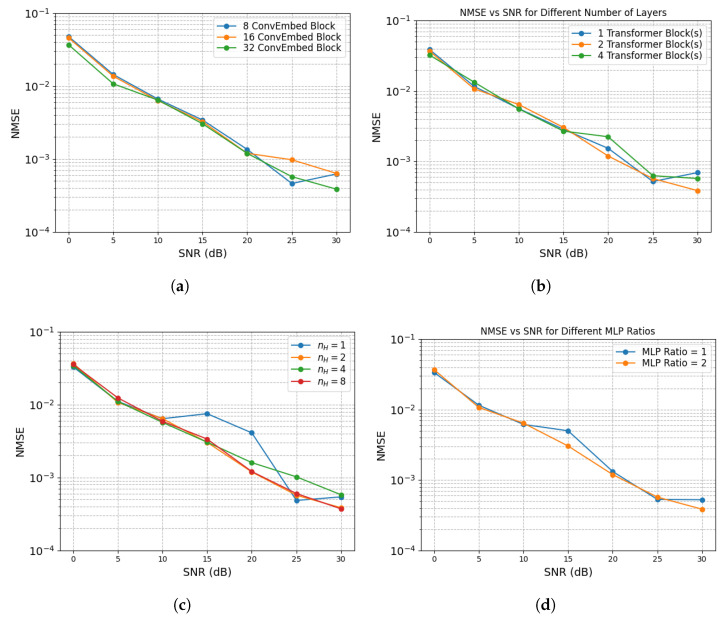
Ablation study in terms of NMSE using various structures with N=25: (**a**) various ConvEmbed blocks; (**b**) various Transformer blocks; (**c**) Various $n_{H}$; (**d**) various MLP expansion ratios.

Generally, the proposed ConvTrans-ResNet consistently outperforms existing methods across a wide range of SNR conditions and IRS configurations. To achieve deeper insight into the contribution of each architectural component, we conduct a comprehensive ablation study by varying key structural parameters. The results are shown in Figure 8.

As shown in Figure 8a, increasing the number of convolutional embedding (ConvEmbed) blocks from 8 to 32 leads to gradual performance improvement, especially under high-SNR scenarios (above 20 dB). The deeper ConvEmbed configurations enhance the model’s ability to extract local spatial features. However, the gain from using 32 blocks over 16 is marginal but requires a higher computational cost. Hence, selecting 16 ConvEmbed blocks is a balanced choice for local feature learning with reasonable efficiency.

In Figure 8b, we investigate the effect of stacking one, two, and four Transformer blocks. Compared to the single Transformer block, using two Transformer blocks yields a more substantial gain by better modeling global dependencies. However, further increasing the number to four leads to diminishing returns and slight degradation at low SNRs, which may be due to overfitting or gradient instability. Therefore, two Transformer blocks are adopted to balance expressiveness and stability.

Figure 8c compares models with varying numbers of attention heads $n_{H}\in \left\{1,2,4,8\right\}$. A single head results in higher NMSE, suggesting that insufficient attention to diversity hinders the model’s ability to learn from heterogeneous spatial patterns. Multiple heads ($n_{H}=2,4,8$) provide better estimation accuracy. However, the performance gain between two and eight heads is negligible, while computational cost increases linearly with $n_{H}$. Thus, $n_{H}=2$ is the best choice to offer a good trade-off between performance and efficiency.

Lastly, Figure 8d analyzes the effect of MLP expansion ratios (1 and 2) in the Transformer feed-forward module. A ratio of 2 marginally improves NMSE at medium-to-high SNRs by increasing hidden dimensionality. Nonetheless, the improvement is minor, and a larger MLP ratio introduces additional parameters. As a result, we choose a ratio of 1, which offers sufficient capacity while maintaining the lightweight nature of the model.

**Figure 9:**
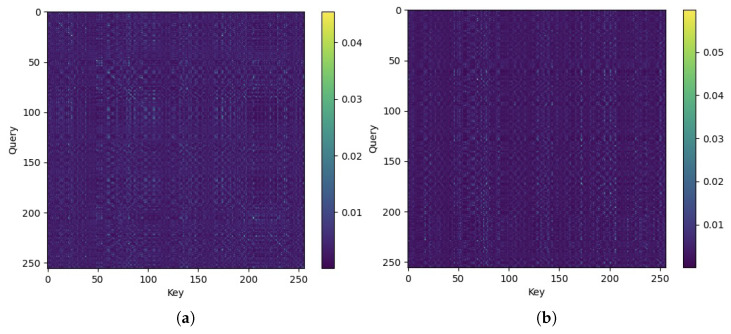
Illustration of attention maps from two attention heads: (**a**) Head 1; (**b**) Head 2.

The advantage of multiple attention heads is that the diversity among heads usually provides complementary perspectives so that a richer representation of the cascaded channel can be learned. The representative attention maps from the Transformer blocks are visualized in Figure 9. Each map shows the learned attention weights between query and key positions for a given sample. It can be observed that the attention mechanism assigns higher weights to a subset of elements while suppressing irrelevant components, which indicates that it selectively captures dominant channel features. It can be confirmed that the attention module effectively models global spatial dependencies and complements the local feature extraction provided by convolutional layers.

In summary, the final architecture is configured with 16 ConvEmbed blocks, two Transformer blocks, two attention heads, and an MLP expansion ratio of 1. This design achieves a favorable balance between performance, computational complexity, and generalization ability, making it well suited for practical deployment in IRS-aided MIMO systems.

### 6.3. Computational Complexity

**Figure 10:**
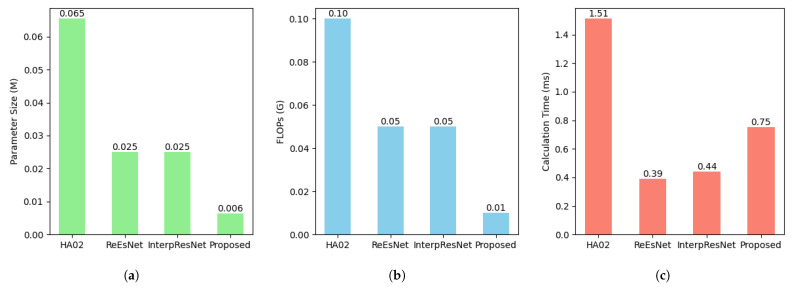
Comparison between various models in terms of complexities: (**a**) parameter size; (**b**) FLOPs; (**c**) calculation time.

The computational complexities of the various methods are assessed from three aspects, including parameter size, floating-point operations (FLOPs), and per-inference calculation time. The comparison is illustrated in Figure 10.

As shown in Figure 10a, our proposed model contains significantly fewer parameters than all other methods. This compact architecture reduces memory consumption and makes the model more suitable for resource-constrained devices. Figure 10b demonstrates that the proposed model achieves the lowest FLOPs with one-fifth of ReEsNet and InterpResNet and one-tenth of HA02. Such a low computational burden highlights the efficiency of our design and confirms its potential for deployment in real-time systems. Although Figure 10c indicates that the inference time of the proposed model is slightly higher than that of ReEsNet and InterpResNet, the model remains substantially faster than HA02. This phenomenon is reasonable since FLOPs and latency are not perfectly correlated. Operations such as multi-head attention and normalization are lightweight in FLOPs but may incur additional memory access and kernel launch overheads. Nevertheless, the proposed model achieves a balanced trade-off by maintaining low overall complexity while incorporating Transformer blocks to capture global dependencies. This modest overhead translates into significant improvements in estimation accuracy, as demonstrated in previous subsections.

Overall, the proposed ConvTrans-ResNet achieves a balance between model size, computational cost, and estimation accuracy. The lightweight design enables fast execution and easy deployment while guaranteeing the robustness and generalizability of accurate channel estimation through attention-based denoising.

## 7. Conclusions and Future Works

In this paper, we propose ConvTrans-ResNet, a lightweight and effective neural network architecture for channel estimation in IRS-aided MIMO systems. By integrating convolutional embeddings and Transformer modules, the model effectively captures both local and global spatial structures within the channel matrix. Furthermore, a residual learning framework is incorporated to enhance training stability and convergence. The proposed network operates on a coarse channel estimate provided by the BALS algorithm. Such a hybrid design benefits from the incorporation of domain knowledge while reducing the overall learning complexity. Extensive experiments were conducted to evaluate the proposed method against state-of-the-art approaches, including HA02, ReEsNet, and InterpResNet. The results demonstrate that ConvTrans-ResNet consistently achieves lower NMSE across a wide range of SNR conditions and IRS configurations, while also maintaining the fewest parameters, the least computational overhead, and a fast inference time.

While the present work assumes block-fading channels, extending the framework to time-varying or mobile scenarios is an important direction. Moreover, applying the framework to multi-user IRS scenarios and jointly optimizing the IRS phase shifts may further improve system-level performance. Since our proposed method is essentially a supervised learning approach, it can be naturally adapted to such cases once reliable ground-truth channel data become available. Possible extensional works include modeling channel dynamics caused by user mobility, Doppler shifts, and temporal fading, as well as incorporating temporal modeling modules to exploit correlations across time. In addition, although computational efficiency has been analyzed in terms of FLOPs and inference latency, future work will also include energy profiling and embedded-device evaluation, which are particularly relevant for IoT and mobile applications. Standard energy-related measures, such as joules per inference and energy per FLOP, will be adopted to provide a fair and comparable assessment of efficiency across different models and hardware platforms.

## Figures and Tables

**Table 1:** Parameters for the simulated MIMO network aided by an IRS.

Symbol	Description	Value
*M*	Number of BS antennas	64
*L*	Number of UE antennas	4
*N*	Number of passive components at IRS	25, 49, 81, 100, 144
$T_{c}$	Channel coherence time	200
*K*	Number of blocks	50
*T*	Number of time slots per block	4
SNR	Signal-to-noise ratios	0:5:30 dB

**Table 2:** Hyperparameters used for training different channel estimation methods.

	HA02	ReEsNet	InterpResNet	Proposed Method
Optimizer	Adam	Adam	Adam	Adam
Maximum epoch	100	100	100	100
Initial learning rate (lr)	0.002	0.001	0.001	0.001
Drop period for lr	every 20	None	every 20	None
Drop factor for lr	0.5	None	0.5	None
Batch Size	128	128	128	128
L2 regularization	1 × 10^−7^	1 × 10^−7^	1 × 10^−7^	1 × 10^−7^

## Data Availability

The original contributions presented in this study are included in the paper.

## Abbreviations

The following abbreviations are used in this manuscript:

IRS	Intelligent Reflective Surface
MIMO	Multiple-Input Multiple-Output
CSI	Channel State Information
DL	Deep Learning
BALS	Bilinear Alternating Least Squares
BS	Base Station
UT	User Terminal
MHSA	Multi-Head Self-Attention
MLP	Multilayer Perceptron
NMSE	Normalized Mean Squared Error
